# Evaluation of professional satisfaction of audiologists working in Türkiye based on their personality characteristics and sector of work

**DOI:** 10.3389/fpsyg.2024.1430118

**Published:** 2024-08-14

**Authors:** Bahtiyar Çelikgün, Furkan Büyükkal, Recep Minga, Gül Ölçek

**Affiliations:** ^1^Department of Audiology, School of Health Sciences, Istanbul Medipol University, Istanbul, Türkiye; ^2^Department of Audiology, Graduate School of Health Sciences, Istanbul Medipol University, Istanbul, Türkiye

**Keywords:** occupational satisfaction, Professional Satisfaction Scale, personality traits, Big Five-50 Personality Traits, audiologists

## Abstract

**Purpose:**

This study investigates the relationship between audiologists' personality traits and professional satisfaction.

**Design:**

Big Five-50 Personality Traits and Professional Satisfaction Scale was used to examine the relationship between audiologists' personality traits and professional satisfaction. Statistical analyzes were performed with SPSS and AMOS programs.

**Study example:**

This study examines 167 audiologists who completed a minimum of an undergraduate program in audiology before the professional field.

**Results:**

The results of confirmatory factor analysis indicate that both scales are reliable and valid. There is a significant relationship between extraversion, agreeableness, conscientiousness, and emotional stability and occupational satisfaction. However, there is no significant relationship between intelligence/imagination and professional satisfaction.

**Conclusions:**

Extraversion, agreeableness, conscientiousness, emotional stability/neuroticism affect job satisfaction. Understanding these dynamics can inform strategies that will increase the professional wellbeing and productivity of audiologists.

## Introduction

The requirement for individuals to work in order to sustain themselves is a phenomenon as ancient as human history. Audiologists, like all other professionals, must work in the fields they find most suitable. Naturally, all of humanity aspires to live a contented life, which is often associated with the concept of “quality of life”, where personal and professional life is balanced. To achieve a sense of overall life satisfaction, individuals must find contentment in both their personal and professional lives (Cimete et al., 2003).

The “job satisfaction theory”, as proposed by Herzberg (2017) in 1959, posits that an individual's level of job satisfaction is contingent upon two distinct factors: hygiene factors, which encompass aspects such as physical working conditions, salary and benefits, and motivation factors, which include factors such as achievement, responsibility and advancement. The theory suggests that job satisfaction is a multifaceted construct, shaped by the interplay of various factors. It has been demonstrated that external factors, such as industry dynamics, and internal factors, such as employees' personality characteristics, can influence the hygiene and motivation-related aspects of occupational satisfaction (Furnham et al., 2002; Faragher et al., 2005).

Audiology is a scientific field that focuses on diagnosing and rehabilitating hearing and balance disorders (Katz, 2009). Graduates of audiology have the opportunity to work in various branches, such as hearing aid sales and application centers, auditory implant companies, audiology clinics in private or public hospitals, auditory rehabilitation centers, and audiology department in universities. It is important to note that each area of audiology has its own unique dynamics and requirements. The specific characteristics of an individual's personality may vary depending on the specific field of audiology in which they are employed. A study revealed a significant correlation between an individual's suitability for their role and their level of professional satisfaction (Li et al., 2023). It is evident that the personality characteristics of audiologists can influence their ability to adapt to the sector in which they work.

Professional satisfaction in audiology may not be achievable in every area for every audiologist due to personality traits. It is important to consider these factors when choosing a career path in audiology. Factors such as introversion/extroversion, adaptability, level of responsibility, emotional stability, openness to new experiences, and imagination may affect an individual's perspective on life and career (Furnham and Zacherl, 1986; Judge et al., 2000, 2002; Törnroos et al., 2019). From another perspective, evaluating the Professional satisfaction of audiologists currently working in different sectors can help identify the needs of audiologists in specific sectors and implement measures to increase Professional satisfaction based on those needs. The purpose of this study is to investigate the correlation between personality traits and Professional satisfaction among audiologists working in various branches of audiology in Türkiye.

## Materials and methods

### Participant

The research sample consisted of 167 audiologists who had completed at least an undergraduate program in audiology and had been employed in a professional setting for at least 3 months. Moreover, the study included audiologists who had graduated from the audiology department but were currently employed in another sector, as well as those who had left their positions despite having a minimum of 3 months of experience. The objective was to assess the compatibility of their personality traits with the sector in which they were working. In contrast, the study excluded individuals who had not yet completed their undergraduate education or had work experience of <3 months.

### Procedure

To investigate the correlation between participants' personality traits and their professional satisfaction, we utilized the Big Five-50 Personality Trait (BFPT) and Professional Satisfaction (PS) Scale. Both surveys were transferred to an online platform using Google Forms. Both tests were prepared on the online platform according to their written versions. The link to the online scales was announced to audiologists working on audiology-related social media channels, accompanied by a social media post explaining the purpose and scope of the study. Participants signed the participant consent form online and completed a short demographic information form consisting of five questions. Next, participants completed two questionnaires: the BFPT, and the PS Scale. Requiring all participants to complete all questions in both surveys eliminated the possibility of any questions being missed or left blank.

The BFPT is a 50-question practical five-point Likert-type assessment tool based on the Five-factor personality model and, examines individuals in terms of five personality traits based on the five-factor personality model: extraversion, agreeableness, conscientiousness, emotional stability/neuroticism, and culture/intelligence/openness to experience or imagination (Digman, 1990; Goldberg, 1992; McAdams, 1992). The test was adapted into Turkish and made available by Tatar (2017). The Turkish version of the test was used in the study (Tatar, 2017).

Additionally, the PS scale consists of 20 five-point Likert-type questions developed from Herzberg's two-factor theory. The primary objective of the scale is to assess the level of Professional satisfaction experienced by an individual in any given profession (Kuzgun et al., 1999). The scale was prepared and validated in 1999 and has been in use since then.

Survey links were activated in September in 2023, based on ethics committee approval, and data collection continued throughout October and November in 2023. At the end of November, survey links were deactivated, and statistical analysis was started.

### Statistical analysis

The study analyzed survey results from 167 participants using SPSS for Windows 25.00 and AMOS 25.0 programs. The sample's demographic characteristics and information about their working experience were presented as percentage rates. Confirmatory factor analyses were conducted for the BFPT and Professional Satisfaction Scale included in the survey form. The study measured the internal consistency, validity, and reliability of the scales by calculating Cronbach's alpha, composite reliability (CR), and average variance explained (AVE) values. Additionally, discriminant validity analysis was conducted to investigate whether the variables were sufficiently separated for path analysis. The study employed a path analysis multiple regression model to examine the impact of the sub-dimensions of the BFPT scale on PS. In addition, the demographic parameters were analyzed using an independent sample *t*-test and one-way ANOVA.

The article was written using the STROBE checklist, and no artificial intelligence support was used at any stage of the study (Von Elm et al., 2007).

## Results

Of the participants, 117 were women (70.1%) and 50 were men (29.9%). The age distribution was as follows: 62 participants (37.1%) were aged 18–24, 87 participants (52.1%) were aged 25–30, seven participants (4.2%) were aged 31–36, four participants (2.4%) were aged 37–42, three participants (1.8%) were aged 43–48, three participants (1.8%) were aged 55–60, and one participant (0.6%) was aged 60 or older. In the study, 126 participants (75.4%) held a bachelor's degree, 35 held a master's degree (21%), and 6 (3.6%) held a doctorate degree (Table 1).

**Table 1:** Demographic information of participants.

		***n***	**%**
Gender	Female	117	70.1%
	Male	50	29.9%
Age groups	18–24	62	37.1%
	25–30	87	52.1%
	31–36	7	4.2%
	37–42	4	2.4%
	43–48	3	1.8%
	55–60	3	1.8%
	60+	1	0.6%
Graduation degree	Bachelor's degree	126	75.4%
	Master's degree	35	21.0%
	Doctorate degree	6	3.6%

Out of all participants, 9% (*n* = 15) stated that they run their hearing aid center, while 34.1% (*n* = 57) worked in the hearing aid sales and application center. In addition, 4.2% (*n* = 7) worked in the auditory implant companies, 11.4% (*n* = 19) worked in Audiology diagnosis clinics in a private or public hospitals, 12% (*n* = 20) worked as academicians in the audiology department of universities, and 16.8% (*n* = 28) worked in auditory rehabilitation centers. Additionally, 11 (6.6%) reported working in a specialist in different fields, while 10 (6%) reported not working in any professional (Table 2).

**Table 2:** Information about the working life of the participants.

		***n***	**%**
Sector you work in?	Work in their hearing aid center	15	9.0%
	Hearing aid sales and application center	57	34.1%
	Implantable hearing solutions brands	7	4.2%
	Audiology diagnosis clinics in a private or state hospital	19	11.4%
	Audiology department in universities	20	12.0%
	Auditory rehabilitation centers	28	16.8%
	A professional other than audiology	11	6.6%
	Not working in any professional	10	6.0%
Working life at the last workplace	0–1 year	67	40.1%
	1–2 years	53	31.7%
	3–5 years	29	17.4%
	5+ years	18	10.8%
Total time spent in business life	0–1 year	32	19.2%
	1–2 years	38	22.8%
	3–5 years	52	31.1%
	5–7 years	19	11.4%
	7 + years	26	15.6%

Table 2 also presents the duration of the participants' total working experience and their working experience at their last workplace. The majority of participants had a working period of 0–5 years in their last workplace (0–1 year: *n* = 67, 40.1%; 1–2 years: *n* = 53, 31.7%; 3–5 years: *n* = 29, 17.4%). Additionally, participants' total time spent in business life was mainly clustered between 0 and 7 years (0–1 year: *n* = 32, 19.2%; 1–2 years: *n* = 38, 22.8%; 3–5 years: *n* = 52, 31.1%; 5–7 years: *n* = 19, 11.4%).

An a priori power analysis was conducted using G^*^Power version 3.1 (Faul et al., 2007) to determine the minimum sample size required to test the study hypothesis. Accordingly, the minimum participant requirement was 159 for the statistics, with an effect size of 0.40, an alpha error of 0.05, and a power ratio of 0.95.

In Confirmatory Factor Analysis, as the sample size increases, the Chi-Square (*x*^2^) value also increases, particularly in samples larger than 200, and the statistical significance level of the Chi-Square (*x*^2^) test decreases (Bollen, 1990). In the evaluation of the scales used for the research and the overall tested models' suitability, Chi-Square (*x*^2^) value corrected by degrees of freedom (Chi-Square value/Degrees of freedom), other goodness of fit indices, and standardized residuals were used. The decision was made after examining the values in the covariance matrix (Bayram, 2010). Goodness of fit indices and fit values used in confirmatory factor analysis are shown in Table 3 (Meydan and Seşen, 2010).

**Table 3:** Goodness of fit indices and fit values used in confirmatory factor analysis.

**Indexes**	**Goodness fit**	**Acceptable fit**	**BFPT**	**PS**
*x*^2^/df	0 ≤ χ^2^/df ≤ 2	2 <χ^2^/df ≤ 3	2.657	2.891
GFI	≥0.90	0.85–0.89	0.934	0.911
CFI	≥0.97	≥0.95	0.954	0.951
SRMR	≤ 0.05	0.06 ≤ SRMR ≤ 0.08	0.065	0.055
RMSEA	≤ 0.05	0.06 ≤ RMSEA ≤ 0.08	0.073	0.069

BFPT, Big five scale; PS, Professional satisfaction scale.

### Confirmatory factor analysis and reliability values for the BFPT

BFPT, which consists of 50 items and five dimensions, underwent confirmatory factor analysis. All items had factor loadings >0.50, indicating that no items were excluded from the analysis. The analysis was conducted using the 50 items as described in the literature, and the standard values of factor loadings ranged from 0.60 to 0.91. The analysis was conducted using the 50 items as described in the literature, and the standard values of factor loadings ranged from 0.60 to 0.91. Based on the model indices presented in Table 3, the BFPT scale demonstrates an acceptable fit. The average variance explained (AVE) value for the BFPT was 0.60, with a CR value of 0.87 and a Cronbach's Alpha value of 0.89, indicating that the scale is both valid and reliable within the sample.

### Confirmatory factor analysis for the PS scale

In the confirmatory factor analysis applied for the PS scale, which has 20 items and one-dimensional, all items were (FY > 0.5), so the item was not eliminated from the analysis. The factor loading standard values ranged from 0.60 to 0.90. Based on the model indices presented in Table 3, the PS Scale demonstrated an acceptable fit.

### Convergent and discriminant validity were applied to the variables included in the model

Composite reliability (CR) values are calculated from factor loadings obtained through confirmatory factor analysis. The combined reliability condition is met when the CR value is ≥0.70 (Raykov, 1997).

The indicator for convergent validity is the AVE value. To confirm convergent validity, the AVE value should be ≥0.50. If the overall composite reliability value (CR) is found to be ≥0.70 and the AVE value is ≥0.40, it is also considered sufficient. To ensure discriminant validity, the square root of the AVE value (√AVE) must be greater than the correlation values in the same row and column (Fornell and Larcker, 1981).

The reliability values of the scales used in the research's first sample were high. Both scales had high reliability values, with 0.929 for the BFPT and 0.911 for the PS scale. All items were found to have high reliability values. The personality traits sub-dimensions were also found to have high reliability values since alpha > 0.80. The reliability condition is met in all sub-dimension variables as the coefficients calculated in all scales in the combined reliability values are (CR ≥ 0.70). Additionally, the necessary condition for convergent validity is met as all variables have average explained variance values (AVE ≥ 0.50). The square root results of the AVE values calculated for discriminant validity are in parentheses. Discriminant validity was achieved since these values were higher than the correlation value in the same row and column (Table 4).

**Table 4:** Convergent and discriminant validity values calculated from standard factor loadings.

**Personality traits**	**Mean**	**SD**	**EI**	**AG**	**CS**	**ES**	**IN**	**PS**
Extraversion-introversion (EI)	2.36	0.71	**(0.775)**					
Agreeableness (AG)	1.81	0.46	0.444^**^	**(0.784)**				
Conscientiousness (CS)	1.81	0.56	0.369^**^	0.434^**^	**(0.777)**			
Emotional stability (ES)	2.78	0.76	0.449^**^	0.253^**^	0.258^**^	**(0.777)**		
Intellect/imagination (IN)	2.00	0.42	0.498^**^	0.377^**^	0.298^**^	0.203^**^	**(0.781)**	
Professional satisfaction (PS)	2.60	0.76	0.458^**^	0.493^**^	0.474^**^	0.425^**^	0.268^**^	**(0.809)**
Cronbach's alpha (CA)			0.813	0.871	0.813	0.811	0.815	0.925
Composite reliability (CR)			0.803	0.865	0.809.	0.806	0.812	0.910
Average variance explained (AVE)			0.602	0.615	0.604	0.605	0.610	0.655

^**^p <0.01.

EI, extraversion-introversion; AG, agreeableness; CS, conscientiousness; ES, emotional stability; IN, intellect/imagination. The bold values represent Discriminant Validity.

### Structural equation modeling path analysis applied with observed values of the research model

**Figure 1:**
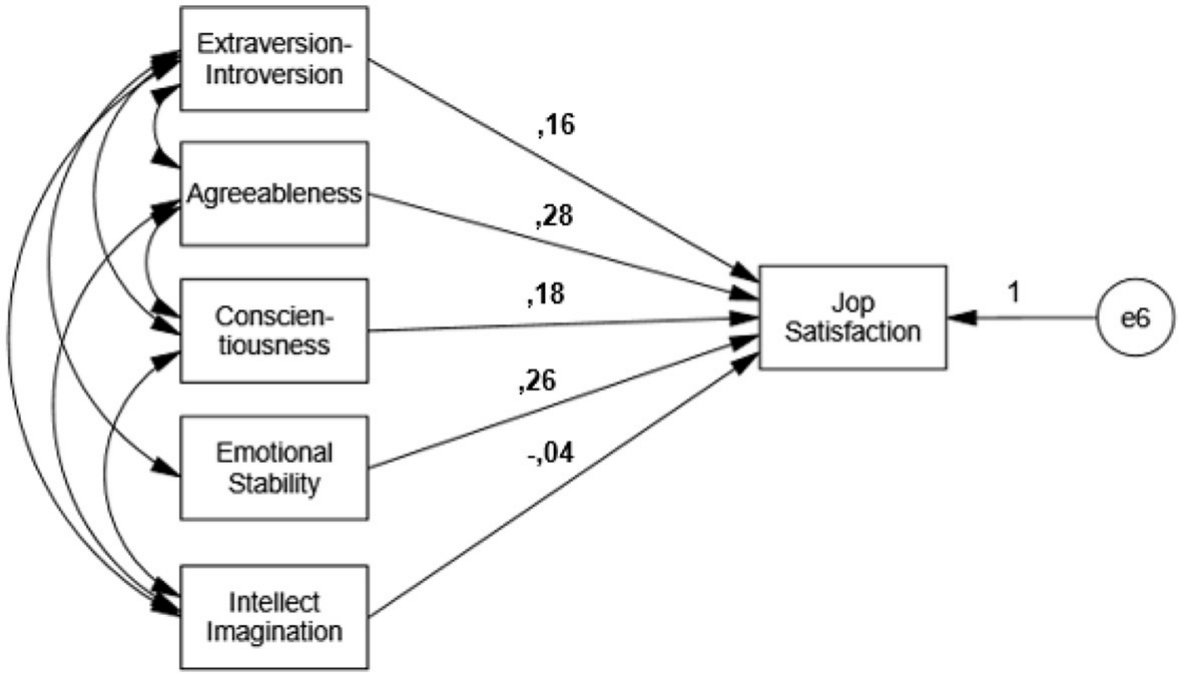
Multiple regression path analysis model with calculated variables.

The path analysis model was used to test the effect of the sub-dimensions of the BFPT scale on the PS variable, using calculated variables. The covariance values found to be significant among the independent variables, which are the sub-dimensions of Personality traits, are also shown (Figure 1).

The model is considered significant based on the model test values of *x*^2^ (8.337), *x*^2^/df (2.779) with a *p*-value of <0.05 in the path analysis model with the observed variables. The model was deemed valid based on the fit index values GFI (0.968), CFI (0.942), SRMR (0.0712), and RMSEA (0.0789), which were within acceptable limits. Table 5 includes the model regression parameters.

**Table 5:** Significance test of regression coefficients in the study.

**Independent**		**Dependent**	**Coefficient**	**CV**	**Z**	**P**	**Hypothesis**
EI	→	PS	0.172	0.161	2.052	0.040^*^	Accepted
AG	→	PS	0.442	0.280	3.794	0.00^**^	Accepted
CS	→	PS	0.336	0.260	3.697	0.00^**^	Accepted
ES	→	PS	0.228	0.237	3.538	0.00^**^	Accepted
IN	→	PS	−0.062	−0.036	−0.492	0.623	Rejected

^**^p <0.01, ^*^p <0.05.

EI, extraversion-introversion; AG, agreeableness; CS, conscientiousness; ES, emotional stability; IN, intellect/imagination.

Table 5 presents the direct regression effects in the multiple regression path analysis model. The effect of the Intellect/Imagination (IN) variable on the Personality traits sub-dimension variables and their effect on the dependent variable of Professional satisfaction was not found to be significant (*p* > 0.05). This effect was considered significant since it was found in all other effects (*p* < 0.05).

The effect of the Extraversion-Introversion variable, one of the personality traits, on the Professional satisfaction variable (β = 0.161; *p* < 0.05) was found to be positive and significant. Accordingly, increasing the Extraversion-Introversion variable also increases the Professional satisfaction variable.

The effect of the Agreeableness variable, one of the personality traits, on the Professional satisfaction variable (β = 0.280; *p* < 0.05) was found to be positive and significant. Accordingly, increasing the Agreeableness variable also increases the Professional satisfaction variable.

The effect of the Conscientiousness variable, one of the personality traits, on the Professional satisfaction variable (β = 0.260; *p* < 0.05) was found to be positive and significant. Accordingly, increasing the Conscientiousness variable also increases the Professional satisfaction variable.

The effect of the Emotional Stability variable, one of the personality traits, on the Professional satisfaction variable (β = 0.237; *p* < 0.05) was found to be positive and significant. Accordingly, increasing the Emotional Stability variable also increases the Professional satisfaction variable.

The effect of the Intellect/Imagination variable, one of the personality traits, on the Professional satisfaction variable (β = −0.036; *p* < 0.05) was not found to be significant. Accordingly, the change in the Intellect/Imagination variable does not affect the Professional satisfaction variable.

### Comparison of variables in the model by sector

A one-way analysis of variance test was used to compare the variables in the model based on the sector worked in Table 6.

**Table 6:** Comparison of the variables in the model by sector of audiology.

**Variables**	**Sector of Audiology**								
	**A**	**B**	**C**	**D**	**E**	**F**	**G**	**H**	***p***
	$\bar{\text{x}}\;\pm$ **SS**	$\bar{\text{x}}\;\pm$ **SS**	$\bar{\text{x}}\;\pm$ **SS**	$\bar{\text{x}}\;\pm$ **SS**	$\bar{\text{x}}\;\pm$ **SS**	$\bar{\text{x}}\;\pm$ **SS**	$\bar{\text{x}}\;\pm$ **SS**	$\bar{\text{x}}\;\pm$ **SS**	
EI	2.4 ± 0.7	2.3 ± 0.7	1.8 ± 0.5	2.7 ± 0.8	2.3 ± 0.8	2.4 ± 0.6	2.6 ± 0.9	2.3 ± 0.8	0.153
AG	1.6 ± 0.4	1.8 ± 0.5	1.8 ± 0.3	2.0 ± 0.5	1.8 ± 0.5	1.8 ± 0.5	1.8 ± 0.5	1.7 ± 0.4	0.402
CS	1.9 ± 0.8	1.8 ± 0.5	1.4 ± 0.5	1.9 ± 0.6	1.8 ± 0.5	1.8 ± 0.6	1.9 ± 0.5	1.9 ± 0.7	0.660
ES	2.4 ± 0.5	2.9 ± 0.8	1.9 ± 0.5	2.8 ± 0.8	2.7 ± 0.7	3.1 ± 0.7	2.7 ± 0.9	2.7 ± 0.6	0.008^**^
IN	1.9 ± 0.4	2.0 ± 0.4	1.7 ± 0.4	2.2 ± 0.3	2.0 ± 0.5	2.0 ± 0.4	2.0 ± 0.7	1.8 ± 0.3	0.267
PS	2.3 ± 0.7	2.6 ± 0.7	2.0 ± 0.8	2.7 ± 0.8	2.3 ± 0.5	2.8 ± 0.6	3.1 ± 1.2	3.0 ± 0.8	0.005^**^

^**^p <0.01.

EI, extraversion-introversion; AG, agreeableness; CS, conscientiousness; ES, emotional stability; IN, intellect/imagination; PS, professional satisfaction.

A: Work in their hearing aid center.

B: Hearing aid sales and application center.

C: Implantable hearing solutions brands.

D: Audiology diagnosis clinics in a private or state hospital.

E: Audiology department in universities.

F: Auditory rehabilitation centers.

G: A Professional other than audiology.

H: Not working in any professional.

**Figure 2:**
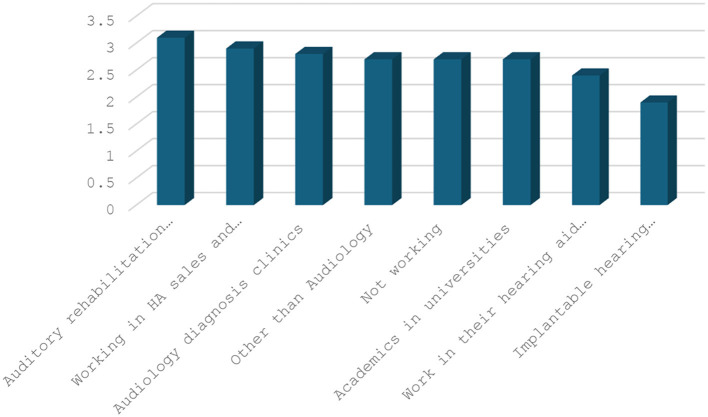
Emotional stability PT averages according to the sector.

**Figure 3:**
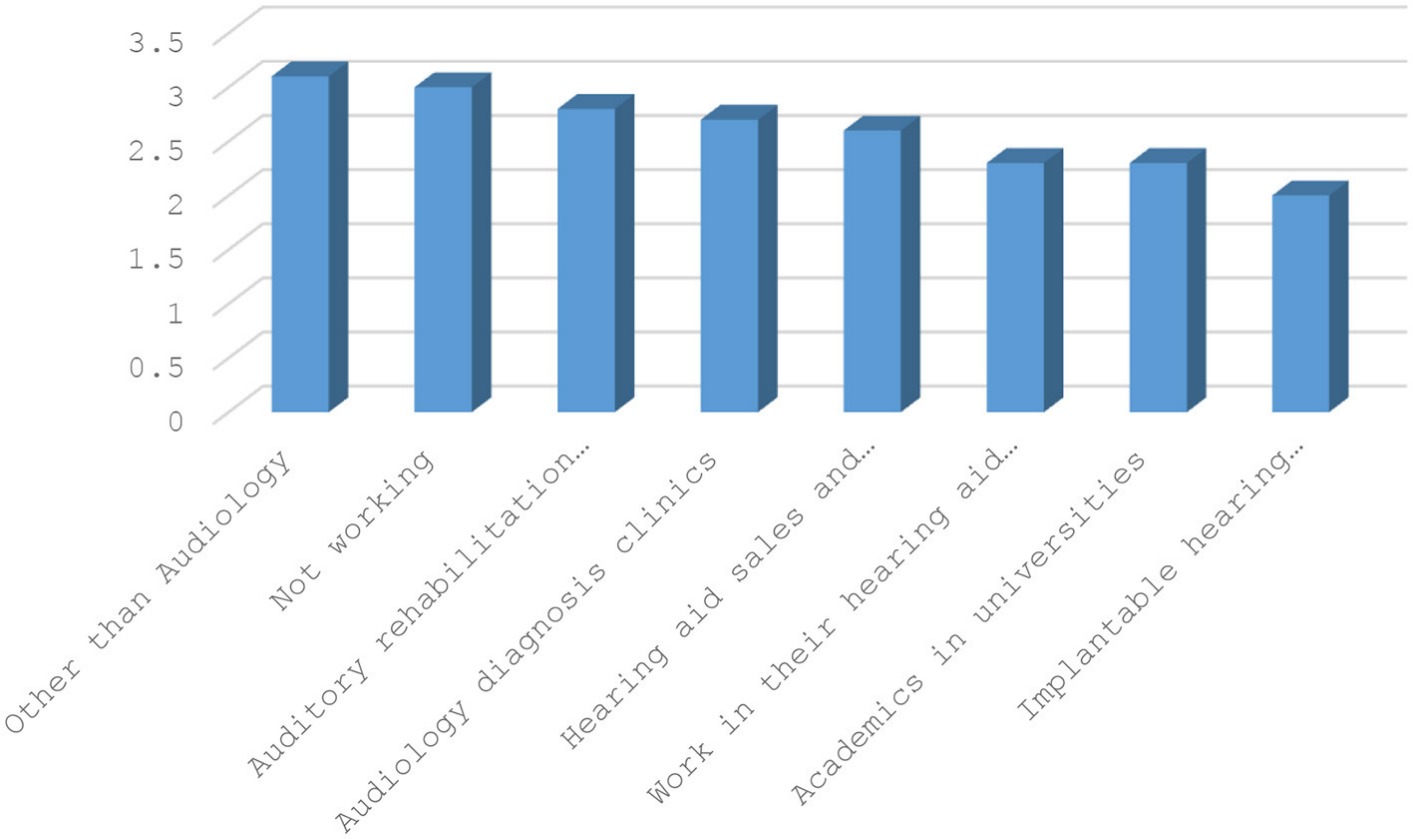
Professional satisfaction averages according to the sector.

Based on the results of multiple comparison tests, it was found that individuals working in special education and rehabilitation centers had a higher average score in the Emotional Stability variable compared to those working in other sectors. Conversely, those working in the cochlear implant sector had a lower average score in the Emotional Stability variable compared to other sectors. Figure 2 shows the average Emotional Stability scores by employment sector. Furthermore, the study found that employees working in sectors other than audiology had a higher average level of Professional satisfaction than employees working in other sectors, and employees working in the cochlear implant sector had a lower average level of Professional satisfaction than employees working in other sectors. Figure 3 shows the average Professional satisfaction variable based on the sector of employment.

### A comparative analysis of job satisfaction and personality traits variables according to demographic characteristics

A comparison of personality trait variables according to gender revealed a significant difference only in the emotional stability trait. Accordingly, it was observed that women exhibited higher scores than men (*p* < 0.001). Upon examination of the variables according to sector of employment, a significant difference was observed only in the variables of professional satisfaction and emotional stability. Therefore, the level of professional satisfaction among audiologists employed in sectors other than audiology is notably higher than that observed among their counterparts in other fields (*p* < 0.05). Upon examination of the emotional stability variable by business sector, it was observed that employees outside the field of audiology exhibited higher emotional stability scores (*p* < 0.05). Furthermore, the comparison of education levels revealed a significant difference in professional satisfaction (*p* < 0.05), while no significant differences were observed in personality traits. Consequently, the highest levels of professional satisfaction were observed among undergraduate graduates.

## Discussion

The objective of the present study is to investigate whether the personality traits of audiologists working in different fields affect their career choice and to examine the relationship between professional satisfaction and audiologists' personality traits. For this purpose, the BFPT and PS scales were used. Naturally first, internal consistency, validity, reliability, confirmatory factor analyses, composite reliability and average variance explained values of the scales were included in the statistical analysis to increase the quality of the study. The analysis revealed that both tests had a high assessment capacity.

The PS scale includes a question about Professional satisfaction for audiologists: “If you were born again, would you like to enter the same profession?” Only 11.4% (*n* = 19) of audiologists answered “always”. In contrast, 25.7% (*n* = 43) answered “never”' while 29.3% (*n* = 49) answered “rarely”. Overall, the PS scale indicated low satisfaction among audiologists with their profession and professional. When examining the distribution of professional satisfaction by field of work, this finding is supported by the fact that those working outside of audiology have higher satisfaction scores. In their 2017 study on professional satisfaction among audiologists in Iran, Mobaraki et al. (2017) found that audiologists reported high levels of professional satisfaction. It is important to consider other factors that may also impact job satisfaction. In 2024, a study found that factors such as self-esteem, age, satisfaction with workplace and working conditions, the effect of workplace on professional development, and income satisfaction have an impact on Professional satisfaction (Cengiz et al., 2024). The participation of primarily young audiologists in our study, the global pandemic, and the considerable economic inflation observed in Türkiye may have had a deleterious impact on the participants' levels of professional satisfaction.

When examining the relationship between Professional satisfaction and personality traits, a positive and significant correlation was found between extraversion personality trait and Professional satisfaction. Specifically, audiologists with extroverted personality traits reported higher Professional satisfaction than introverts. It should be noted that there is no consensus on this issue in the literature. For example, Chandrasekara's (2019) study found that all personality traits have an impact on Professional satisfaction. Similarly, a study conducted in China with over 800 participants found a significant correlation between extraversion and wellbeing, as well as Professional satisfaction (Zhai et al., 2013). In contrast, a comprehensive study conducted by Bui (2017), which included 7,662 participants, found no correlation between extroverted personality and professional satisfaction. This difference may be related to the sectors and professions in which individuals work or may depend on the country of residence.

The study found a positive and significant correlation between Professional satisfaction and the Agreeableness personality trait. Audiologists with higher agreeableness scores reported higher professional satisfaction. Similar to other personality traits, there is no consensus on agreeableness in the literature. Our finding is consistent with Chandrasekara's (2019) research, which identified agreeableness as the personality trait that most strongly influences Professional satisfaction. On the other hand, Petasis and Economides (2002) found no relationship between personality traits, including agreeableness, and professional satisfaction.

In the current study, it was found that the conscientiousness personality trait has a positive effect on Professional satisfaction. Audiologists with high conscientiousness scores experienced higher Professional satisfaction. Topino et al. (2021) also reported a strong relationship between conscientiousness and professional satisfaction. Bowling (2010) found a moderate relationship between the conscientiousness personality trait and Professional satisfaction in his study. It is important to note that this relationship is not definitive and further research is needed to fully understand the connection between these two variables.

Similar to other personality traits, a positive relationship was found between emotional stability and Professional satisfaction. The study found that audiologists with higher emotional stability scores reported higher Professional satisfaction levels. Frye (2000) conducted a study that examined the relationship between emotional stability and Professional satisfaction. Based on the study results, emotional stability has both direct and indirect causal effects on Professional satisfaction, with the indirect effect being slightly higher than the direct effect. Porwal and Sharma (1985) discussed these two elements from a different perspective and stated that individuals with high Professional satisfaction exhibited more emotional consistency. Additionally, the field of study may affect the emotional stability of audiologists, even if they share the same profession. Our study found that audiologists in the field of auditory rehabilitation exhibit higher emotional stability values than average, while those in the field of implantable hearing solutions exhibit lower values.

Finally, the relationship between the Intellect/Imagination personality trait and Professional satisfaction was examined in this study. Unlike other personality traits, no significant relationship was observed. Therefore, the Professional satisfaction variable is not affected by changes in the Intellect/Imagination score. Michels (2022) conducted a study examining the relationship between Professional satisfaction and personality traits. The study found no relationship between any personality trait, including Intellect/Imagination, and Professional satisfaction. However, Bui (2017) and Chandrasekara (2019) explained that there is a significant relationship between the Intellect/Imagination trait and Professional satisfaction similar to other personality traits.

The findings of our study indicate that women exhibit greater emotional stability than men. The results of a study conducted with university students in 2022 indicated that there was no statistically significant relationship between emotional stability and gender (Ahmed and Çerkez, 2020). Nevertheless, a study conducted with 2,500 employees in Newport, England, indicated that men exhibited greater emotionality than women in the workplace (Simpkin, 2019). The study revealed that men were twice as likely as women to engage in emotionally volatile behavior at work, including yelling and quitting. This finding is consistent with the results of our study.

A further issue that emerged from our study is that job satisfaction is subject to variation according to the level of education attained. The participants in our study who had obtained an undergraduate degree indicated that they experienced a higher level of job satisfaction than those who had completed a master's or doctoral degree. This finding is consistent with the education-employment fit theory, which is widely accepted in the academic literature (Vila and García-Mora, 2005). Consequently, individuals who are unable to perform the duties of a position commensurate with their educational qualifications tend to exhibit low levels of job satisfaction, as they perceive a lack of opportunity to fully utilize their abilities. In our study, the inverse correlation between education level and job satisfaction may be related to this phenomenon. Moreover, it is known that there is a significant positive correlation between job satisfaction and salary (Al-Zoubi, 2012). The Turkish economy has recently experienced inflation and a general decline in salaries, which may have contributed to lower job satisfaction among Audiology employees compared to those in other fields.

Although there is no definitive consensus in the literature, a large body of research suggests a significant relationship between personality traits and Professional satisfaction. Other factors that may affect Professional satisfaction include profession, cultural background, stress levels, self-esteem, age, workplace satisfaction, working conditions, professional development opportunities, and income (Cengiz et al., 2024). It is important to consider all of these factors when evaluating Professional satisfaction. Therefore, future studies should take a holistic approach to measuring audiologists' Professional satisfaction to obtain more comprehensive data.

The study included audiologists who were primarily between the ages of 25 and 30 and employed at hearing centers. It is acknowledged that this situation represents a limitation of the study. Nevertheless, this may be attributed to the fact that the inaugural cohort of undergraduate audiology students in Türkiye graduated in 2015, and there are more employment opportunities in hearing centers compared to public and private hospitals. We strongly believe that our study sample accurately represents the current state of audiology in Türkiye. Furthermore, the disproportionate representation of participants in the field of audiology was identified as a potential limitation of the study. To address this, appropriate statistical techniques were employed to substantiate the findings.

For our study, evaluating only personality traits using a scale can be seen as an area for development. However, a comprehensive personality trait test and thorough statistical analysis of all scales used in the study are strengths of the research.

## Conclusions

The study findings suggest that Professional satisfaction among audiologists in Türkiye is generally low. Audiologists working outside of audiology-related fields report higher professional satisfaction than those working within the field. Additionally, audiologists working in the field of implantable hearing solutions report the lowest levels of professional satisfaction. The examination of the relationship between personality traits and Professional satisfaction revealed a significant and positive correlation between Professional satisfaction and all personality traits, except for Intellect/Imagination.

As the inaugural study on job satisfaction in the field of audiology, the study presents significant findings for both employers and audiology associations in Türkiye. It is a significant finding that, while a considerable proportion of individuals employed in various fields of audiology report below-average levels of job satisfaction, those working outside of audiology tend to exhibit higher levels of job satisfaction. Discussions held at congresses and other meetings involving collaboration between audiology associations, universities, and private sector entities may prove instrumental in addressing this issue.

## Data availability statement

The original contributions presented in the study are included in the article/supplementary material, further inquiries can be directed to the corresponding author.

## Ethics statement

The studies involving humans were approved by Istanbul Medipol University Ethics Committee. The studies were conducted in accordance with the local legislation and institutional requirements. The participants provided their written informed consent to participate in this study.

## Author contributions

BÇ: Writing – original draft, Writing – review & editing, Investigation, Methodology, Project administration, Supervision, Conceptualization, Data curation, Formal analysis, Resources, Validation. FB: Conceptualization, Data curation, Formal analysis, Investigation, Methodology, Resources, Software, Validation, Visualization, Writing – review & editing. RM: Conceptualization, Data curation, Formal analysis, Investigation, Resources, Software, Validation, Visualization. GÖ: Writing – review & editing.
